# Association of *ACE2* variant rs4646188 with the risks of atrial fibrillation and cardioembolic stroke in Uygur patients with type 2 diabetes

**DOI:** 10.1186/s12872-021-01915-9

**Published:** 2021-02-18

**Authors:** Cheng Liu, Jingxian Pei, Yanxian Lai, Tianwang Guan, Abudurexiti Zeyaweiding, Tutiguli Maimaiti, Haiyan Zhao, Yan Shen

**Affiliations:** 1 Department of Cardiology, Guangzhou First People’s Hospital, South China University of Technology, 1 Panfu Road, Guangzhou, 510180 China; 2 Department of Cardiology, The Second Affiliated Hospital of Guangzhou Medical University, Guangzhou, 510260 China; 3 Department of Cardiology, Guangzhou First People’s Hospital, Guangzhou Medical University, Guangzhou, 510180 China; 4 Department of Cardiology, Shufu People’s Hospital, Kashgar Region, 844100 Xinjiang Uygur Autonomous Region (XUAR) China

**Keywords:** Angiotensin-converting enzyme 2, Polymorphism, Atrial fibrillation, Cardioembolic stroke, Type 2 diabetes, Uygurs

## Abstract

**Background:**

Atrial fibrillation (AF) is the most common cardiac arrhythmia. Type 2 diabetes (T2D) is an independent risk factor for AF. The cardioembolic stroke (CS) risk is increased when both conditions coexist. Whether angiotensin-converting enzyme 2 (ACE2) genetic variants predict increased risks AF and CS in Uygur patients with T2D remain elusive.

**Methods:**

A total of 547 Uygur subjects (272 controls and 275 T2D patients) were recruited to the study from south Xinjiang. Eight ACE2 variants were identified by MassARRAY system.

**Results:**

ACE2 rs2074192 (CC, adjusted RR = 2.55, 95% CI 1.35–4.80, *P* = 0.004), rs4240157 (CC + CT, adjusted RR = 2.26, 95% CI 1.27–4.04, *P* = 0.006) and rs4646188 (TT, adjusted RR = 2.37, 95% CI 1.16–4.86, *P* = 0.018) were associated with higher AF risk. ACE2 rs4240157 (CC + CT, adjusted RR = 2.68, 95% CI 1.36–5.27, *P* = 0.004) and rs4646188 (TT, adjusted RR = 2.56, 95% CI 1.06–6.20, *P* = 0.037) were further associated with higher CS risk. The 3 ACE2 variants were related to larger left atrial end-systolic diameter (LAD) (all *P* < 0.05), but not all of the 3 ACE2 variants were related to increased levels of serum sodium (rs4240157 and rs4646188, all *P* < 0.05), HsCRP (rs4240157 and rs4646188, all *P* < 0.05) as well as decreased serum potassium levels (rs2074192 and rs4646188, all *P* < 0.05). The 3 ACE2 variants exhibited heterogeneity on circulating RAAS activation. In particular, ACE2 rs4646188 was associated with higher levels of ACE (*P* = 0.017 and 0.037), Ang I (*P* = 0.002 and 0.001), Ang II (both *P* < 0.001) and ALD (*P* = 0.005 and 0.011).

**Conclusion:**

These results indicated *ACE2* rs4646188 was associated with increased risk of AF and CS among diabetic patients in Uygurs, which could be a promising genetic predisposition marker for early and personalized prevention strategies for the aforementioned clinical pathologies.

## Background

Atrial fibrillation (AF) is the most common arrhythmia worldwide, and type 2 diabetes (T2D) is an independent risk factor for AF [1]. The exact mechanisms underlying the increased risk of AF in T2D patients are not fully elucidated, but are thought to involve structural and electrical remodeling of atria, inflammation, oxidative stress, and glycemic fluctuation [2]. Currently, the prevalence of diabetes in China has rapidly increased over the past decade, and has been ranked top one around the world [3]. The incidence of AF is then increasing with the diabetes epidemic. More Studies indicated that the risk of cardioembolic stroke (CS) is increased when both conditions coexist, becoming a major public health problem in China. Although optimal glycemic management and integrated control of these reversible risk factors (e.g., unhealthy diet, smoking, physical inactivity, obesity, and obstructive sleep apnea syndrome, dyslipidemia, etc.) have been related to lower incidence of AF and CS, the benefit could be dampened or offset by inherently greater genetic risk [4]. Here, early genetic risk evaluation of individual’s at greater risk of AF and CS will provide a possible strategy for precision prevention of AF and CS in patients with T2D.

It is well known that the occurrence and development of T2D is associated with renin–angiotensin–aldosterone system (RAAS) activation [5], and through entire chain of diabetic related cardiovascular events (e.g., AF [6]). Angiotensin-converting enzyme 2 (*ACE2*), as a key negative regulator of the RAAS, hydrolyze respectively angiotensin I/II (Ang I/II) into Ang (1–9)/Ang (1–7), and Ang (1–7) further acts on the Mas receptor, forming the ACE2/Ang(1–7)/Mas axis. The axis not only maintains blood glucose homeostasis via improving sensitivity to insulin but also promotes cardiovascular protection by vasodilation, anti-proliferation, anti-oxidative stress, anti-inflammatory, anti-fibrosis, and anti-thrombosis, which against the biological effects of the classic ACE/Ang II/AT1R axis [7]. In experimental AF models, the expression of atrial *ACE2* was decreased in association with overexpression of atrial Ang II and the development of atrial structural remodeling [8]. In contrast, atrial overexpression of *ACE2* improved cardiac fibrosis, electrical remodeling, and its related AF inducibility [9]. These results suggested that ACE2 now has become a new target for prevention of AF and CS.

ACE2 is one of the major candidate genes for AF genetic susceptibility. The ACE2 gene sequence is highly polymorphic and high degree of genetic heterogeneity, manifesting the characteristics of geographical-, ethnic- and gender-specific genetic diversity [10]. Many studies showed the relationship between ACE2 SNPs and the upstream cause of AF as mentioned above. Patel et al*.* [11] reported that 3 *ACE2* variants (e.g., rs2074192, rs4240157 and rs4646188) were related to the risk of diabetes related HTN in Australian Caucasians. Chaoxin et al*.* reported that *ACE2* rs2285666 variant was associated with CAD among patients with T2D in South China [12]. Recent study by Liu et al*.* [13] confirmed that 8 *ACE2* SNPs (e.g., rs233575, rs879922, rs2074192, rs1978124, rs4646188, rs2048683, rs4646156 and rs4240157) were not only linked to moderate to high risk of T2D but also with higher risk of hypertension and dyslipidemia in Uygur patients with T2D. These results suggested that there was a potential link between ACE2 variants and AF due to the common genetic basis. Indeed, Feng et al*.* [14] observed that with *ACE2* rs6632677 increased the structural AF risk in male Han Chinese, which 15.7% participants in the study had diabetes. Wang et al*.* [15] also found that the male Han Chinese carrying T-allele of *ACE2* rs2106809 was is associated with lone AF and adverse cardiac events (e.g., stroke). Luo et al*.* [10] reported that 3 *ACE2* SNPs (e.g., rs4646155, rs4240157 and rs4830542) were correlated with the risk of hypertension induced AF in South Xinjiang. However, the relationship of ACE2 gene polymorphism with AF and CS is not fully understood in Uygur patients with T2D. Accordingly, in present study we evaluated the relationships of *ACE2* gene variants with AF and CS among diabetic patients in Uygurs.

## Methods

### Study participants

The ethics approval (2014SYYLSZ-018) of this study was granted from Guangzhou First People’s Hospital, South China University of Technology. A total of 547 subjects were enrolled from south Xinjiang of China, and were divided into two groups: T2D (N = 275) and non-T2D (N = 272) group. All adult (age ≥ 18 years) participants were diagnosed at baseline with T2D referring to the American Diabetes Association (ADA) recommendations [16]: (1) a random serum glucose levels ≥ 11.1 mmol/L in a participant with symptoms of marked hyperglycemia including polyuria, polydipsia, polyphagia and weight loss; (2) a fasting plasma glucose (FPG) levels ≥ 7.0 mmol/L; (3) 2 h plasma glucose levels ≥ 11.1 mmol/L via oral glucose tolerance test (OGTT); (4) plasma glycosylated hemoglobin (HbA1C) concentration ≥ 6.5%. The non-T2D participants who underwent medical examination at the same hospital during the same period were enrolled as the control group. Any participants combined with HTN, CAD, HF, AF and stroke at the enrollment were ruled out from the study. Standard analytical methods were performed to assess blood biochemical indexes on admission to the study. Echocardiogram was carried out on enrollment to the study referring to relevant recommendations [17].

### Long-term follow-up endpoint

Primary follow-up endpoint was new-onset AF. AF was newly diagnosed referring to prior medical record or 12-lead electrocardiogram reports from enrollment to follow-up. The newly diagnosed AF was defined as those who present for the first time with persistent or paroxysmal AF during regular follow-up period, regardless of whether the duration of the arrhythmia is known at the time of presentation. The cardiac thrombosis was evaluated according to the ASE’s guidelines [18]. Stroke was defined as the presence of a focal/global neurological event with symptoms and signs lasting > 24 h. All stroke subjects were survivors of CS, and determined by magnetic resonance image and/or computed tomography scanning of the brain referring to relevant guidelines [19]. All participants were followed up since the date of the enrollment. The date of final follow-up was Dec 31, 2019.

### Genotyping assay processing

Eight *ACE2* variants were genotyped using the Sequenom MassARRAY system using our previously described methods [20], was shown Additional file 1: Table S1. Primer software (Version 5.0, Cambridge, USA) was used to design the specific primers for the 8 *ACE2* single nucleotide polymorphism (SNPs) based on the *ACE2* gene sequence information in GenBank, was shown in Additional file 1: Table S2. The specific primers of *ACE2* SNPs were composited by Invitrogen (Guangzhou, China). The SNPs determination accuracy was 100% for each variant of *ACE2*.

### Statistical analysis

The softwares of SPSS (version 24, SPSS Co., USA) and PASS (version 15, Statistical Solutions Ltd, Ireland) were used for statistical analyses. The Hardy–Weinberg equilibrium was only analyzed with χ2 test for female participants, due to *ACE2* gene is on the X chromosome. Continuous variables were showed as mean ± SD while categorical variables were as number (percentage). The independent sample t test and two-way analysis of variance (ANOVA) were carried out to assess the significant differences for continuous variables. The χ2 test was carried out to evaluate the associations of *ACE2* mutations with those categorical variables. Binary logistic regression analysis was executed to evaluate the relative risk (RR) of AF as well as CS. Bonferroni correction was carried out to adjust the probability of type I error (false positive). A *P* value < 0.05 is statistically significant. All probabilities are two-tailed.

## Results

### Characteristics of the study subjects

Baseline clinical profiles of the study participants are listed in Additional file 1: Table S3. There were significant differences on levels of blood pressure (e.g., SBP and DBP), BMI, blood lipid (e.g., TRIG and HDL-C), blood sugar (e.g., FBG and HbA1C), blood uric acid, serum sodium/potassium, MAU and RAAS (e.g., renin and Ang II) between the two groups (all *P* < 0.05). After an average 6.8-years follow-up, no participants were dropped, the cumulative incidence of AF and CS is 16.1% and 8.8%, respectively. Subjects with or without diabetes had obviously differences on medical condition and use of antihypertensive drugs, including HTN (*P* = 0.001), CAD (*P* = 0.014), AF (*P* = 0.006), CS (*P* < 0.001), calcium channel blockers (CCBs, *P* = 0.018), beta-receptor blockers (BBs, *P* = 0.011), and renin-angiotensin system inhibitors (RSIs, *P* = 0.002), as shown in Additional file 1: Table S4.

### Association between *ACE2* variants and new-onset AF in diabetic participants

3 *ACE2* variants were correlated with higher risk of AF in participants with T2D during follow-up period as follows: rs2074192 (CC, adjusted RR = 2.55, 95% CI 1.35–4.80, *P* = 0.004), rs4240157 (CC + CT, adjusted RR = 2.26, 95% CI 1.27–4.04, *P* = 0.006), rs4646188 (TT, adjusted RR = 2.37, 95% CI 1.16–4.86, *P* = 0.018), as shown in Table 1. This phenomenon was only observed in the subgroup of participants with T2D. In subgroup of participants without T2D, none of the eight ACE2 variants was associated with AF risk (all adjusted *P* > 0.05).

**Table 1:** Association between *ACE2* SNPs and new-onset AF in diabetic participants

*ACE2* SNPs	Total				Non-T2D subgroup				T2D subgroup			
	Non-AF (N/%)	AF (N/%)	RR (95%CI)*	*P* value*	Non-AF (N/%)	AF (N/%)	RR (95%CI)^#^	*P* value ^#^	Non-AF (N/%)	AF (N/%)	RR (95%CI)^#^	*P* value ^#^
*rs1978124*												
*CC*	277 (60.3)	54 (61.4)	1.00		158 (65.8)	16 (50.0)	1.00		119 (54.3)	38 (67.9)	1.00	
*TT* + *CT*	182 (39.7)	34 (38.6)	0.76 (0.42–1.38)	0.364	82 (34.2)	16 (50.0)	1.34 (0.43–4.12)	0.616	100 (45.7)	18 (32.1)	0.66 (0.27–1.63)	0.369
*rs2048683*												
*GG*	329 (71.7)	64 (72.7)	1.00		196 (81.7)	26 (81.2)	1.00		133 (60.7)	38 (67.9)	1.00	
*TT* + *GT*	130 (28.3)	24 (27.3)	0.66 (0.34–1.26)	0.207	44 (18.3)	6 (18.8)	0.30 (0.06–1.56)	0.151	86 (39.3)	18 (32.1)	1.07 (0.39–2.97)	0.896
*rs2074192*												
*CC*	190 (41.4)	50 (56.8)	2.55 (1.35–4.80)	0.004	94 (39.2)	10 (31.3)	0.44 (0.13–1.51)	0.191	96 (48.3)	40 (71.4)	3.22 (1.26–8.24)	0.015
*TT* + *CT*	269 (58.6)	38 (43.2)	1.00		146 (60.8)	22 (60.7)	1.00		123 (56.2)	16 (28.6)	1.00	
*rs233575*												
*CC* + *CT*	142 (30.9)	28 (31.8)	0.99 (0.53–1.86)	0.983	48 (20.0)	10 (31.3)	1.59 (0.54–4.68)	0.397	94 (42.9)	18 (32.1)	0.70 (0.18–2.66)	0.598
*TT*	317 (69.1)	60 (68.2)	1.00		192 (80.0)	22 (60.7)	1.00		125 (57.1)	38 (67.9)	1.00	
*rs4240157*												
*CC* + *CT*	140 (30.5)	48 (54.5)	2.26 (1.27–4.04)	0.006	54 (22.5)	10 (31.3)	0.97 (0.31–3.06)	0.960	86 (39.3)	38 (67.9)	3.38 (1.15–9.98)	0.027
*TT*	319 (69.5)	40 (45.5)	1.00		186 (77.5)	22 (60.7)	1.00		133 (60.7)	18 (32.1)	1.00	
*rs4646156*												
*AA* + *AT*	134 (29.2)	24 (27.3)	0.61 (0.32–1.16)	0.134	48 (20.0)	6 (18.8)	0.29 (0.06–1.47)	0.134	86 (39.3)	18 (32.1)	0.79 (0.28–2.24)	0.659
*TT*	325 (70.8)	64 (72.7)	1.00		192 (80.0)	26 (81.2)	1.00		133 (60.7)	38 (67.9)	1.00	
*rs4646188*												
*CC* + *CT*	152 (33.1)	14 (15.9)	1.00		88 (36.7)	8 (25.0)	1.00		64 (29.2)	6 (10.7)	1.00	
*TT*	307 (66.9)	74 (84.1)	2.37 (1.16–4.86)	0.018	152 (63.3)	24 (75.0)	1.17 (0.28–4.92)	0.827	155 (70.8)	50 (89.3)	3.03 (1.06–8.66)	0.039
*rs879922*												
*CC* + *CG*	244 (53.2)	60 (68.2)	0.95 (0.52–1.75)	0.878	114 (47.5)	14 (43.8)	0.92 (0.35–2.37)	0.857	130 (59.4)	46 (82.1)	1.03 (0.35–3.00)	0.957
*GG*	215 (46.8)	28 (31.8)	1.00		126 (52.5)	18 (56.3)	1.00		89 (40.6)	10 (17.9)	1.00	

* Model 1: after adjustment for age, HT, T2D, LDL-C and LAD

# Model 2: It is the same as Model 1 with exception of T2D

### Association between *ACE2* variants and CS in diabetic participants

*ACE2* rs4240157 (CC + CT, adjusted RR = 2.68, 95% CI 1.36–5.27, *P* = 0.004), rs4646188 (TT, adjusted RR = 2.56, 95% CI 1.06–6.20, *P* = 0.037) were correlated with higher risk of CS in participants with T2D during follow-up period, as shown in Table 2.

**Table 2:** Association between *ACE2* SNPs and incident CS in diabetic participants

*ACE2* SNPs	CS (N/%)		RR (95% CI)*	*P* value*
	NO	YES		
*rs2074192*				
CC	214 (42.9)	26 (54.2)	1.26 (0.63–2.54)	0.515
TT + CT	285 (57.1)	22 (45.8)	1.00	
*rs4240157*				
CC + CT	160 (32.1)	28 (58.3)	2.68 (1.36–5.27)	0.004
TT	339 (67.9)	20 (41.7)	1.00	
*rs4646188*				
CC + CT	159 (31.9)	7 (14.6)	1.00	
TT	340 (68.1)	41 (85.4)	2.56 (1.06–6.20)	0.037

* Model 3: after adjustment for age, HT, AF, LDL-C and LAD

### *ACE2* variants and the size of left atrial end-systolic diameter (LAD)

T2D subjects with the high AF risk genotype of *ACE2* rs2074192 (CC, *P* = 0.001 and < 0.001), rs4240157 (CC + CT, *P* = 0.006 and < 0.001), rs4646188 (TT, both *P* < 0.001) were further correlated with larger size of LAD, as shown in Table 3.

**Table 3:** The size of left atrial end-systolic diameter in relation to different *ACE2* SNPs among participants with or without T2D

*ACE2* SNPs	LAD (cm)		
	Non-T2D	T2D	*P* value
*rs2074192*			
*CC*	2.82 ± 0.29	2.93 ± 0.41	0.016
*TT* + *CT*	2.90 ± 0.33	3.10 ± 0.41	< 0.001
*P* value	0.063	0.001	
*rs4240157*			
*CC* + *CT*	2.85 ± 0.30	3.09 ± 0.31	< 0.001
*TT*	2.87 ± 0.32	2.96 ± 0.48	0.059
*P* value	0.716	0.006	
*rs4646188*			
*CC* + *CT*	2.86 ± 0.33	2.83 ± 0.36	0.632
*TT*	2.87 ± 0.31	3.07 ± 0.41	< 0.001
*P* value	0.674	< 0.001	

### *ACE2* variants and serum sodium/potassium levels

T2D subjects with the high AF risk genotype (TT) of *ACE2* rs4646188 was correlated with increased serum sodium level (*P* = 0.001 and < 0.001) and lower potassium concentration (both *P* < 0.001). However, *ACE2* rs4240157 (CC + CT, *P* = 0.027 and 0.003) was only associated with increased serum sodium level while rs2074192 (CC, *P* = 0.014 and 0.001) were only associated with lower potassium concentration, as shown in Table 4.

**Table 4:** Serum sodium/potassium levels in relation to different *ACE2* SNPs among participants with or without T2D

*ACE2* SNPs	Serum Sodium (Na^+^, mmol/L)			Serum Potassium (K^+^, mmol/L)		
	Non-T2D	T2D	*P* value	Non-T2D	T2D	*P* value
*rs2074192*						
*CC*	138.6 ± 3.7	140.2 ± 5.8	0.010	4.22 ± 0.25	4.12 ± 0.22	0.001
*TT* + *CT*	139.5 ± 3.5	140.7 ± 5.3	0.021	4.23 ± 0.32	4.20 ± 0.30	0.415
*P* value	0.052	0.468		0.949	0.014	
*rs4240157*						
*CC* + *CT*	139.2 ± 3.8	141.2 ± 4.8	0.003	4.21 ± 0.26	4.15 ± 0.25	0.091
*TT*	139.1 ± 3.5	139.8 ± 6.1	0.223	4.23 ± 0.30	4.17 ± 0.28	0.049
*P* value	0.895	0.027		0.707	0.522	
*rs4646188*						
*CC* + *CT*	139.4 ± 3.5	138.3 ± 6.0	0.221	4.20 ± 0.30	4.27 ± 0.24	0.104
*TT*	139.0 ± 3.6	141.1 ± 5.3	< 0.001	4.24 ± 0.29	4.13 ± 0.26	< 0.001
*P* value	0.370	0.001		0.271	< 0.001	

### *ACE2* variants and serum HsCRP levels

T2D subjects with the high AF risk genotypes of rs4240157 (CC + CT, *P* = 0.005 and 0.036) and rs4646188 (TT, *P* < 0.001 and = 0.001) was correlated with higher level of HsCRP, as shown in Table 5.

**Table 5:** Serum HsCRP levels in relation to different *ACE2* SNPs among participants with or without T2D

*ACE2* SNPs	HsCRP (mg/L)		
	Non-T2D	T2D	*P* value
*rs2074192*			
*CC*	12.5 ± 13.1	11.7 ± 13.1	0.637
*TT* + *CT*	11.0 ± 11.4	13.3 ± 14.5	0.126
*P* value	0.309	0.342	
*rs4240157*			
*CC* + *CT*	11.5 ± 9.5	15.2 ± 13.8	0.036
*TT*	11.6 ± 12.8	10.4 ± 13.6	0.387
*P* value	0.944	0.005	
*rs4646188*			
*CC* + *CT*	11.7 ± 10.1	7.8 ± 5.3	0.001
*TT*	11.5 ± 13.2	13.9 ± 15.2	0.095
*P* value	0.853	< 0.001	

### *ACE2* variants and serum RAAS levels

In the comparison between the high AF risk and control genotypes of the AF risk related *ACE2* SNPs, there were significant differences on serum concentration of RAAS activation as follows: ACE [rs4646188 (*P* = 0.017 and 0.037)], Ang I [rs4240157 (both *P* < 0.001) and rs4646188 (*P* = 0.002 and 0.001)], Ang II [rs2074192 (both *P* < 0.001), rs4240157 (both *P* < 0.001), rs4646188 (both *P* < 0.001)], and ALD [rs2074192 (both *P* < 0.001) and rs4646188 (*P* = 0.005 and 0.011)], as shown in Table 6.

**Table 6:** Serum RAAS levels in relation to different *ACE2* SNPs among participants with or without T2D

*ACE2* SNPs	ACE (U/L)			Renin (pg/mL)			Ang I (ng/L)		
	Non-T2D	T2D	*P* value	Non-T2D	T2D	*P* value	Non-T2D	T2D	*P* value
*rs2074192*									
CC	40.3 ± 13.3	40.1 ± 15.6	0.936	33.2 ± 24.6	44.1 ± 18.9	< 0.001	2.39 ± 0.84	2.68 ± 1.02	0.015
TT + CT	40.9 ± 8.1	43.0 ± 14.4	0.137	32.7 ± 24.1	47.4 ± 23.4	< 0.001	2.48 ± 0.85	2.68 ± 1.12	0.081
*P* value	0.656	0.118		0.868	0.189		0.389	0.995	
*rs4240157*									
CC + CT	38.9 ± 10.8	41.1 ± 15.4	0.243	34.5 ± 19.9	44.9 ± 21.4	0.001	2.37 ± 0.92	2.94 ± 0.97	< 0.001
TT	41.2 ± 10.2	41.9 ± 14.8	0.628	32.4 ± 25.5	46.5 ± 20.2	< 0.001	2.47 ± 0.82	2.47 ± 1.11	0.949
*P* value	0.117	0.682		0.501	0.539		0.449	< 0.001	
*rs4646188*									
CC + CT	41.5 ± 11.4	37.6 ± 17.0	0.113	33.7 ± 24.1	45.2 ± 20.7	0.002	2.43 ± 0.69	2.32 ± 1.00	0.450
TT	40.2 ± 9.7	42.7 ± 14.2	0.037	32.5 ± 24.4	45.9 ± 20.8	< 0.001	2.45 ± 0.93	2.79 ± 1.07	0.001
*P* value	0.306	0.017		0.704	0.814		0.788	0.002	

*ACE2* SNPs	Ang II (ng/L)			ALD (ng/L)					
	Non-T2D	T2D	*P* value	Non-T2D	T2D	*P* value			
*rs2074192*									
CC	116.6 ± 62.1	162.9 ± 55.0	< 0.001	243.1 ± 73.8	224.7 ± 101.1	0.105			
TT + CT	126.2 ± 81.3	135.1 ± 46.5	0.232	235.1 ± 82.5	276.9 ± 110.8	< 0.001			
*P* value	0.305	< 0.001		0.419	< 0.001				
*rs4240157*									
CC + CT	118.9 ± 65.6	168.7 ± 46.8	< 0.001	233.2 ± 83.2	259.9 ± 107.5	0.085			
TT	123.7 ± 77.2	132.6 ± 51.7	0.193	239.6 ± 78.1	243.8 ± 110.2	0.688			
*P* value	0.655	< 0.001		0.574	0.225				
*rs4646188*									
CC + CT	127.4 ± 85.5	117.5 ± 48.5	0.345	244.5 ± 76.4	222.2 ± 85.6	0.082			
TT	119.6 ± 67.2	158.3 ± 50.2	< 0.001	234.3 ± 80.9	259.9 ± 114.0	0.011			
*P* value	0.406	< 0.001		0.306	0.005				

## Discussion

AF and CS among patients with T2D are a growing public health issues that has received increasing attention. Current treatment and prevention strategies for AF among T2D patients are mainly focused on glucose-lowering therapies, restoration and maintenance of sinus rhythm (e.g., drugs, cardioversion or catheter ablation), heart rate control and stroke prevention [2]. The occurrence of AF and CS can be reduced by treatment by those measures for T2D patients. However, as far as the early prevention and control of AF and CS are concerned, clinical evaluation of genetic risk is an important link in comprehensive management of AF in diabetes that cannot be ignored [21]. Like diabetes, recent genetic studies indicated that there is also an obvious genetic predisposition for the occurrence of AF [22]. It is noted that the prevalence of T2D in China has the characteristics of ethnic diversity (e.g., Uygur) [23], and there may also be ethnic differences in AF. Thus, early identification and assessing the heritability of AF will provide a novel possible strategy for the prevention and control of AF among T2D patients in Uygurs.

This is the only study to investigate possible relationships between *ACE2* polymorphisms and AF among Uygur patients with T2D. Our previous research reported that there are 8 *ACE2* variants related to the risk of diabetes [13]. However, the data showed in this study suggest that only the diabetes risk related *ACE2* rs4646188 was further correlated with high risk AF (RR = 2.37, Table 1) and CS (RR = 2.58, Table 2) in participants with T2D via a median follow-up of 6.8 years. Indeed, Patel et al*.* [11] reporting that Caucasian T2D patients carrying TT genotype of the loci was associated HTN. Pan et al*.* [20] recently report that subjects carrying T-allele of *ACE2* rs4646188 were not only related to high HTN risk but also to increased dyslipidemia risk include elevated serum levels of triglyceride (TRIG), total cholesterol (TC) and low-density lipoprotein cholesterol (LDL-C) as well as decreased serum high-density lipoprotein cholesterol (HDL-C). *ACE2* rs4646188 was not only linked to with the upstream causes of AF (e.g., HTN, dyslipidemia and diabetes) but also to the downstream complications (e.g., stroke [10]). Similar phenomenon were also seen at the other *ACE2* SNP such as rs2285666, which was linked to higher HTN [24] and stroke [25] risk in northern Han Chinese, but the loci seem to play lesser roles in T2D [26] and lone AF [15] risk. These results suggested that there is ethnic difference between Uygurs and Hans in the relationship between *ACE2* variants and AF.

It is well known that atrial structural and electrical remodeling are important elements for the occurrence and maintenance of AF. The macroscopic phenotype of atrial structural remodeling is left atrial enlargement (LAE), which has been considered to be an independent predictor of AF [27]. In Coronary Artery Risk Development in Young Adults (CARDIA) [28], the presence of T2D at baseline was related to larger LAD via a 20-year follow-up. In this study we found that the only 3 T2D risk related *ACE2* mutations (e.g., rs2074192, rs4240157 and rs4646188) were further correlated with increased LAD in T2D patients (Table 3 and Additional file 1: Table S5). Our results are partially accordant with a recent studies as follows: rs2074192 was associated with left ventricular remodeling in hypertensive patients [29], and rs4240157 was linked to left ventricular remodeling among Austrian in MONICA Augsburg Echocardiographic Substudy [30]. The *ACE2* rs4240157 was also correlated with larger LAD in Uygur patients with HTN while rs4646188 was not [10]. What’s more important, LAE is an independent predictor of stroke and systemic embolism in patients with AF or in sinus rhythm [31, 32].

Besides atrial structural remodeling, atrial electrical remodeling also acts a primary role in occurrence and development of AF. The mechanism of atrial electrical remodeling can be divided into heterogeneity of action potential duration (APD) and electrical conduction abnormalities, involving in specific ion channels remodeling (e.g., sodium and potassium), which were affected by changes in serum sodium [33, 34] and potassium [35] concentrations. In this study we also found not all AF risk related *ACE2* mutations were related to changes of serum sodium/potassium concentrations. *ACE2* rs4240157 was only linked to elevated serum sodium levels among in Uygur patients with T2D, and rs2074192 was only associated with decreased serum potassium levels. Uygur patients with high diabetes risk genotype of *ACE2* rs4646188 were associated with both the higher serum sodium and lower serum potassium levels. Indeed, studies have reported that elevated serum sodium levels and hypokalemia are a risk factor for postoperative atrial fibrillation [36]. The other 4 diabetes risk related *ACE2* variants (*e,g.*, rs2074192, rs2048683, rs4646156 and rs879922) were not related to AF risk. T2D subjects with the high diabetes risk genotypes of the 4 *ACE2* mutations were associated with higher serum sodium levels it had nothing to do with lower serum potassium levels (Additional file 1: Table S6). These phenomena indicated that the simultaneous changes of serum sodium/potassium concentration is more conducive to the occurrence of AF under the ACE2 mutations related genetic background, and the changes in serum potassium may be more important than changes in serum sodium for AF susceptibility. Our results suggested that *ACE2* rs4646188 could be involved in atrial electrical remodeling by mediating the changes of serum sodium/potassium concentrations, thereby leading to T2D patients present with an increased susceptibility to AF.

Numerous evidences have implicated inflammation as central mediators of T2D induced atrial structural and electrical remodeling. HsCRP, as a key biomarker of inflammation, has been not only related to diabetes but also to the occurrence, maintenance and recurrence of AF [37] as well as left atrial thrombus formation [38]. However, clinical trials on evaluating the efficacy of anti-inflammatory drugs (e.g., colchicine, corticosteroids, statins and omega-3 unsaturated fatty acids) in prevention of AF were inconsistent [39]. Besides the differences in anti-inflammatory drugs used, these findings indicate that it is essential to determine appropriate populations (high inflammation risk) with anti-inflammatory treatment. In present study we further found that T2D subjects carrying the high diabetes and AF risk genotype of rs4646188 had significantly increased plasma HsCRP levels (Table 5) and CS risk. In addition, T2D subjects with the greater diabetes risk genotypes of *ACE2* rs1978124 had higher inflammation level (Additional file 1: Table S7) and CS risk, but it was not related to AF risk. These results indicated that inflammation acts an important role for occurrence and development of AF from the perspective of atrial remodeling and its induced thrombus formation, but the ionic basis (especially serum potassium) of maintain AF trigger AF seems more important.

Even though a poor understanding of the exact molecular mechanisms of *ACE2* variants on increased AF risk in patients with diabetes, accumulated evidence suggested that activation of RAAS triggers a series of events (e.g., left atrial remodeling, inflammation and thromboembolism) contributing to AF. The common clinical profile (e.g., obesity, dyslipidemia and HTN, etc.) of the T2D subjectscreates the perfect environment for AF to occur, and the essence is metabolic disorder [40], which is the initiating factor for RAAS activation [41]. Diabetes induced activation of the ACE/Ang II/AT1R axis can promote cardiomyocyte and endothelial cell apoptosis, fibrosis, oxidative stress generation and inflammation [41], that leads to cardiac remodeling and creates a favorable condition for AF occurrence. *ACE2* is an important component of RAAS, may be equally or even more important than ACE in regulating the cardiac balance of the RAAS, particularly in the setting of diabetes [42]. Indeed, recent study indicated that increased ACE2 serum level was linked to higher risk of total death, cardiovascular events (e.g., myocardial infarction, heart failure and stroke) and diabetes [43]. The role of *ACE2* variants in AF remains unclear, and it could be assumed that it is due to the ACE2/ACE axis imbalance. *ACE2* deficiency leads to increased tissue and circulating levels of Ang II under status of T2D [44]. *ACE2* SNPs rs2106809 and rs2074192 have been associated with reduced circulating Ang (1–7) [45]. These results suggested that the *ACE2* SNPs may directly or indirectly result in circulating activation of RAAS. Our findings found that *ACE2* rs4646188 were significantly linked to RAAS activation (e.g., ACE, ANG I/ II and ALD), partially consistent with previous research.

### Limitations

The major disadvantages of the study were as follows: firstly, due to the sample size (N = 547), large-scale subgroup analysis based on comparing the intensity of association between diabetic and non-diabetic patients will further demonstrate the importance of ACE2 in T2D patients. Secondly, Bonferroni correction was executed to correct significance thresholds, but false-positive results may still occur. Thirdly, the complex associations between genotype (ACE2 variants) and phenotype (AF and its related CS) to ultimately predict trait heritability remains a primary challenge of modern genetics. The exosome-derived microRNAs (exo-miRs) are one of the main classes of non-coding RNAs, and play a critical role as bridge that links genetic and environment factors. Functional characterization of the effects of genetic variants on differentially expressed exo-miRs related signaling pathways will undoubtedly facilitate our understanding of the associations between genotypes and phenotypes, needing further research. Therefore, results must be interpreted carefully.

### Future directions

*ACE2* variants rs4646188 could be a promising genetic predisposition marker for the risk of AF and CS among diabetic patients in Uygurs. Early genotype identification of the loci could provide a time window for targeted prevention and treatment of AF and CS in Uygur diabetes with large-scale prospective study.

## Supplementary Information

**Additional file 1**. Association of* ACE2* variant rs4646188 with the risks of atrial fibrillation and cardioembolic stroke in Uygur patients with type 2 diabetes.

## Data Availability

All of the data are available with reasonable request from the corresponding author.

## Abbreviations

ACE2: Angiotensin converting enzyme 2
AF: Atrial fibrillation
Alb: Albumin
ALD: Aldosterone
ALT: Alanine aminotransferase
Ang I/ II: Angiotensin I/II
AST: Aspartate aminotransferase
BMI: Body mass index
BUN: Blood urea nitrogen
CAD: Coronary atherosclerotic heart disease
Cr: Creatinine
CS: Cardioembolic stroke
DBP: Diastolic blood pressure
FPG: Fasting plasma glucose
HF: Heart failure
HTN: Hypertension
HbA1C: Glycosylated hemoglobin
HDL-C: High-density lipoprotein cholesterol
HsCRP: Hypersensitive C-reactive protein
LAD: Left atrial end-systolic diameter
LAE: Left atrial enlargement
LDL-C: Low-density lipoprotein cholesterol
Lp(a): Lipoprotein A
MAU: Microalbumin in urine
RAAS: Renin–angiotensin–aldosterone system
SBP: Systolic blood pressure
SNP: Single nucleotide polymorphism
T2D: Type 2 diabetes
TC: Total cholesterol
TRIG: Riglyceride
UA: Blood uric acid
