# Supplementary material for: Association of ACE2 variant rs4646188 with the risks of atrial fibrillation and cardioembolic stroke in Uygur patients with type 2 diabetes
Source: BMC Cardiovasc Disord. 2021 Feb 18;21:103. doi: 10.1186/s12872-021-01915-9 (PMC7890811; doi:10.1186/s12872-021-01915-9)
Supplement: Supplementary file 1 — Additional file 1. Association of ACE2 variant rs4646188 with the risks of atrial fibrillation and cardioembolic stroke in Uygur patients with type 2 diabetes. [file 12872_2021_1915_MOESM1_ESM.docx]

**Supplementary material**

**Materials and methods**

**Sample collection and** **genotyping assay processing**

Peripheral venous whole blood samples were collected into anticoagulation tube with EDTA on enrollment. Hemolysis was assessed according to the previously reported method, and hemolyzed samples were excluded from the experimental workflow. The extraction of genomic DNA was used the Blood DNA extraction kit (Invitrogen, California, USA) according to the manufacturer's protocol, and diluted to 10 ng/μL after quantification by NanoDrop-1000 (ThermoFisher, Waltham, MA). 8 *ACE2* variants (*e.g.*, rs1978124, rs2048683, rs2074192, rs233575, rs4240157, rs4646156, rs4646188 and rs879922) were genotyped using the Sequenom MassARRAY system according to the manufacturer's instructions with minor modifications based on literatures and human genome sequence databases [1], was shown Table S1. Primers for the 8 *ACE2* SNPs were designed by Primer 5.0 (Whitehead Institute Cambridge, Massachusetts, USA) based on the *ACE2* gene sequence in GenBank [*e.g.*, NC_000023.11:g.15599940T>C; NC_000023.11:g.15590376T>G; NC_000023.11:g.15564667C>T; NG_012575.1:g.42227C>T; NC_000023.11:g.15568841C>T; NC_000023.10:g.15597043A>T; NG_012575.1:g.23850T>C; NG_012575.1:g.34386G>C], was shown in Table S2. The primers were synthesized by Invitrogen Life Science Technologies (Guangzhou, China). The accuracy of the genotypes determined was 100% for each *ACE2* SNP.

**Cardiac ultrasonography**

Cardiac ultrasonic scanning was performed when participants admitted to the study. The left atrial end-diastolic dimension (LAD) were measured using M-mode or two-dimensional echocardiography in the parasternal long-axis view at the end-ventricular systole with a 1.7/3.4 MHz linear array transducer (Vivid 7, GE Healthcare, USA) over 4 cardiac cycles according to recommendations for chamber quantification from the American Society of Echocardiography [2]. The cardiac thrombosis was also evaluated according to the ASE’s guidelines [3].

**Statistical analysis**

All analysis was using SPSS version 24 (SPSS, Chicago, IL) and PASS version 15 (Statistical Solutions Ltd, Cork, Ireland). The Hardy-Weinberg equilibrium was only analyzed with a Chi-square test for female participants, due to *ACE2* gene is on the X chromosome. Categorical variables are presented as frequencies including gender, smoking, drinking, T2D and AF. The Chi-square test was used to analyze the associations of *ACE2* SNPs with those categorical variables. Binary logistic regression analysis was used to evaluate the relative risk (RR) of AF and cardioembolic stroke. Given that false positive might exist in the results, Bonferroni adjustment was used to adjust the significance thresholds for multinomial logistic regression. Continuous variables are presented as the mean ± SD, including age, systolic blood pressure (SBP), diastolic blood pressure (DBP), body mass index (BMI), blood biochemical index [*e.g.*, white blood cell count (WBC), alanine aminotransferase (ALT), creatinine (Cr), hypersensitive C-reactive protein (HsCRP), triglyceride (TRIG), high-density lipoprotein cholesterol (HDL-C), microalbumin in urine (MAU) and Ang II, etc] and echocardiography index [*e.g.*, left atrial end-systolic diameter (LAD)], etc. The independent sample t test and two-way analysis of variance (ANOVA) were used to analyze the significant differences for continuous variables. *P* values of less than 0.05 were considered as statistically significant. All probabilities are two-tailed estimated.

**Results**

***ACE2* SNPs and genotype frequencies**

Not all the 8 *ACE2* SNPs examined were in Hardy-Weinberg equilibrium (HWE, *P*>0.05), and heterogeneity was observed in study participants (see Supplemental Table S1). In non-T2D participants only 4 *ACE2* SNPs [*e.g.*, rs1978124 (*P*=0.129), rs4646156 (*P*=0.155), rs4646188 (*P*=0.202) and rs879922 (*P*=0.405)] were in HWE.

***ACE2* SNPs and the size of left atrial end-systolic diameter**

As shown in Table S5, significant difference were observed between the T2D and non-T2D participants on LAD size (all *P*<0.05), but no significance were observed between the high diabetes risk and control genotypes of diabetes risk related *ACE2* SNPs rs2074192, rs2048683, rs233575, rs4646156 and rs879922 (all *P*>0.05).

***ACE2* SNPs and serum sodium/****potassium levels**

As shown in Table S6, T2D patients with the high diabetes risk genotypes of *ACE2* rs2074192 (TT+CT, *P*=0.024 and <0.001), rs2048683 (TT+CT, both *P*=0.001), rs4646156 (AA+AT, *P*=0.001 and =0.004) and rs879922 (CC+CG, *P*=0.013 and =0.002) except rs233575 (CC+CT, *P*=0.404 and =0.095) were associated with increased serum sodium level. However, the 5 diabetes risk related *ACE2* SNPs were not associated with lower potassium concentration. Interestingly, the serum potassium levels in T2D patients with the high diabetes risk genotypes of rs233575 (CC+CT, *P*=0.004) and rs879922 (CC+CG, *P*=0.004) were higher than that with its corresponding control genotype despite there was no significant difference between T2D and non-T2D participants with the same high diabetes risk genotypes of the 2 ACE2 variants (*P*= 0.474 and 699, respectively).

***ACE2* SNPs and serum HsCRP levels**

As shown in Table S7, T2D patients with the high diabetes risk genotypes of *ACE2* rs2074192 (TT+CT, *P*=0.021 and =0.023) with exception of rs2048683, rs233575, rs4646156 and rs879922 (all *P*>0.05).

**Supplemental Table legends**

Table S1. Descriptive information on *ACE2* SNPs in study participants

Table S2. *ACE2* SNP primers used in the Sequenom MassARRAY system

Table S3. Baseline characteristics of study participants

Table S4. Partial baseline characteristics of study participants at the end of the follow-up

Table S5. The size of left atrial end-systolic diameter in relation to different ACE2 SNPs among participants with or without T2D.

Table S6. Serum sodium/potassium levels in relation to different ACE2 SNPs among participants with or without T2D.

Table S7. Serum HsCRP levels in relation to different ACE2 SNPs among participants with or without T2D.

**Table S1. Descriptive information on *ACE2* SNPs in study participants**

| **NQ** | ***ACE2* SNPs** | **MAF in**  **CHB/CEU*** | **Major/minor allele** | **MAF** | | **Power** | ***P_HWE_*-value^#^** |
| --- | --- | --- | --- | --- | --- | --- | --- |
|  |  |  |  | **Non-T2D** | **T2D** |  |  |
| 1 | *rs1978124* | 0.006/0.483 | *C/T* | 0.195 | 0.364 | 0.998 | 0.129 |
| 2 | *rs2048683* | 0.006/0.409 | *G/T* | 0.125 | 0.262 | 0.852 | 0.019 |
| 3 | *rs2074192* | 0.463/0.349 | *C/T* | 0.478 | 0.316 | 0.999 | 0.022 |
| 4 | *rs233575* | 0.006/0.362 | *T/C* | 0.136 | 0.269 | 0.915 | <0.001 |
| 5 | *rs4240157* | 0.044/0.383 | *T/C* | 0.162 | 0.327 | 0.981 | 0.033 |
| 6 | *rs4646156* | 0.006/0.403 | *T/A* | 0.129 | 0.255 | 0.878 | 0.155 |
| 7 | *rs4646188* | 0.000/0.087 | *T/C* | 0.301 | 0.160 | 0.999 | 0.202 |
| 8 | *rs879922* | 0.044/0.389 | *G/C* | 0.279 | 0.422 | 0.999 | 0.405 |

*MAF: minor allele frequency; CHB：Han Chinese in Beijing, China；CEU：Utah residents with Northern and Western

European ancestry

# *P*_HWE_ value for non-T2D (control) participants

**Table S2. The primers of *ACE2* SNPs in the Sequenom MassARRAY system**

| **NQ.** | **SNP_ID** | **2nd-** **reverse PCR primer (5’-3’)** | **1st-** **forward PCR primer (5’-3’)** | **extension primer (5’-3’)** |
| --- | --- | --- | --- | --- |
| 1 | *rs1978124* | *ACGTTGGATGGAGAGAACTTTGGAAACCTG* | *ACGTTGGATGAAGCTGCTGATGTAGAAGTG* | *CCATATCTCTATCTGATGGAC* |
| 2 | *rs2048683* | *ACGTTGGATGCAGTGAACATGGCAGTGTAG* | *ACGTTGGATGTGATCCAGCAATCCCTCTTC* | *GGCAGTGTAGATATCTTTATGAAG* |
| 3 | *rs2074192* | *ACGTTGGATGTTAGGTTCATCAACAGCTCC* | *ACGTTGGATGCCCTTAAACACAGCAGTCAC* | *CAAGGGTGGAAATGTATAAATGGTTGG* |
| 4 | *rs233575* | *ACGTTGGATGAGGTCCTATGACCAAGTCTC* | *ACGTTGGATGTTTCTTATGTGCCTCCCCAG* | *TCCTATGACCAAGTCTCTATAGTA* |
| 5 | *rs4240157* | *ACGTTGGATGTTGCTCAGTGAATTGGCCTC* | *ACGTTGGATGTTTCCATGCAGTGAGGGTTG* | *CTCAGAACATTACAGAATCAAAC* |
| 6 | *rs4646156* | *ACGTTGGATGGGGAAATAGATATGATGGGC* | *ACGTTGGATGCCTTAGGTACTTGGACCTTC* | *GGGCCATGGAACAGG* |
| 7 | *rs4646188* | *ACGTTGGATGGATATTCTACTCAGAAACG* | *ACGTTGGATGCTCTGTGTTCCCTTCTGTTG* | *GGGGAACGTAGAATTTTAGTTGAATG* |
| 8 | *rs879922* | *ACGTTGGATGGCTCCAGCAAATTCAAGGAC* | *ACGTTGGATGGGCAGTTTATTGTACATTGTG* | *CTCAAGGACTGGGGTTA* |

**Table S3. Baseline characteristics of study participants**

|  | **Non-T2D** | **T2D** | ***P* value** |
| --- | --- | --- | --- |
| Sample (N) | 272 | 275 | - |
| Follow-up time (Y) | 6.7±0.6 | 6.7±0.8 | 0.938 |
| Male: Female | 110:162 | 95:180 | 0.154 |
| Age (Y) | 58.1±13.0 | 59.2±10.5 | 0.248 |
| Smoking (%) | 55(20.2) | 68(24.7) | 0.207 |
| Drinking (%) | 38(14.0) | 51(18.5) | 0.147 |
| SBP(mmHg) | 115.7±9.5 | 124.2±11.5 | <0.001 |
| DBP(mmHg) | 75.5±8.3 | 81.2±9.6 | <0.001 |
| BMI (Kg/m^2^) | 25.7±4.1 | 28.0±3.8 | <0.001 |
| TRIG (mmol/L) | 1.33±0.49 | 1.63±0.51 | <0.001 |
| TC (mmol/L) | 4.74±0.90 | 4.87±1.20 | 0.129 |
| HDL-C (mmol/L) | 1.23±0.17 | 1.18±0.15 | <0.001 |
| LDL-C (mmol/L) | 2.80±0.61 | 2.88±0.64 | 0.124 |
| Lp-A (g/L) | 0.24±0.14 | 0.20±0.13 | 0.001 |
| ApoA1/ApoB | 1.39±0.26 | 1.41±0.35 | 0.281 |
| FPG (mmol/L) | 5.57±0.41 | 6.83±0.71 | <0.001 |
| HbA1C (%) | 5.59±0.59 | 6.57±0.84 | <0.001 |
| Cr (μmol/L) | 69.9±23.6 | 67.3±22.4 | 0.195 |
| BUN (mmol/L) | 5.13±1.77 | 5.14±1.78 | 0.977 |
| UA (μmol/L) | 270.4±91.1 | 328.9±86.5 | <0.001 |
| ALT (U/L) | 25.0±13.3 | 23.2±12.0 | 0.111 |
| AST (U/L) | 24.4±10.9 | 24.1±9.5 | 0.687 |
| Alb (g/L) | 39.5±3.3 | 39.5±8.7 | 0.939 |
| Na^+^ (mmol/L) | 139.1±3.6 | 140.4±5.6 | 0.001 |
| K^+^ (mmol/L) | 4.23±0.29 | 4.16±0.27 | 0.005 |
| HsCRP (mg/L) | 11.6±12.3 | 12.6±17.3 | 0.418 |
| ACE (U/L) | 40.7±10.4 | 41.6±15.1 | 0.421 |
| Renin (pg/mL) | 32.9±24.3 | 45.8±20.8 | <0.001 |
| Ang I (ng/L) | 2.44±0.85 | 2.48±1.07 | 0.004 |
| Ang II (ng/L) | 122.5±74.5 | 148.9±52.6 | <0.001 |
| ALD (ng/L) | 238.9±79.3 | 251.1±109.1 | 0.136 |

**Table S4. Partial baseline characteristics of study participants at the end of the follow-up**

|  | **Non-T2D** | **T2D** | ***P* value** |
| --- | --- | --- | --- |
| **Sample(N)** | **272** | **275** | **-** |
| **Medical condition** |  |  |  |
| HTN | 89(32.7) | 128(46.5) | 0.001 |
| CAD (%) | 76(27.9) | 104(37.8) | 0.014 |
| AF (%) | 32(11.8) | 56(20.4) | 0.006 |
| Stroke (%) | 12(4.4) | 36(13.1) | <0.001 |
| **NYHA** |  |  |  |
| I | 87(32.0) | 78(28.4) | 0.457 |
| II | 131(48.2) | 127(46.2) |  |
| III | 43(15.8) | 56(20.3) |  |
| IV | 11(4.0) | 14(5.1) |  |
| **Combined medication** |  |  |  |
| (A)Antiplatelet drugs | 123(45.2) | 134(48.7) | 0.411 |
| (B)Warfarin | 15(5.5) | 18(6.5) | 0.613 |
| (C) Statins | 143(52.6) | 162(58.9) | 0.136 |
| (D) RSIs | 66(24.3) | 101(36.7) | 0.002 |
| (E) BBs | 46(16.9) | 71(25.8) | 0.011 |
| (F) MRA | 51(18.8) | 64(23.5) | 0.172 |
| (G) CCBs | 58(21.3) | 83(30.2) | 0.018 |
| (H) Diuretics | 34(12.5) | 41(14.9) | 0.413 |
| (J) Nitrates | 26(9.6) | 40(14.5) | 0.073 |

**Table S5. The size of left atrial end-systolic diameter in relation to different ACE2 SNPs among participants with or without T2D.**

| ***ACE2* SNPs** | | **LAD(cm)** | | |
| --- | --- | --- | --- | --- |
|  |  | **Non-T2D** | **T2D** | ***P* value** |
| *rs1978124* | *CC* | 2.88±0.32 | 2.98±0.33 | 0.005 |
|  | *TT+CT* | 2.85±0.31 | 3.05±0.48 | <0.001 |
|  | *P* value | 0.511 | 0.138 |  |
| *rs2048683* | *GG* | 2.86±0.32 | 3.04±0.39 | <0.001 |
|  | *TT+GT* | 2.89±0.31 | 2.98±0.45 | 0.151 |
|  | *P* value | 0.540 | 0.300 |  |
| *rs233575* | *CC+CT* | 2.82±0.25 | 2.97±0.39 | 0.002 |
|  | *TT* | 2.88±0.33 | 3.04±0.43 | <0.001 |
|  | *P* value | 0.124 | 0.158 |  |
| *rs4646156* | *AA+AT* | 2.88±0.31 | 2.98±0.45 | 0.093 |
|  | *TT* | 2.86±0.32 | 3.04±0.39 | <0.001 |
|  | *P* value | 0.748 | 0.315 |  |
| *rs879922* | *CC+CG* | 2.88±0.34 | 3.03±0.42 | 0.001 |
|  | *GG* | 2.85±0.29 | 2.98±0.41 | 0.007 |
|  | *P* value | 0.422 | 0.320 |  |

**Table S6. Serum sodium/potassium levels in relation to different ACE2 SNPs among participants with or without T2D.**

| ***ACE2* SNPs** | | **Serum Sodium (Na^+^, mmol/L)** | | |  | **Serum Sodium (K^+^, mmol/L)** | | |
| --- | --- | --- | --- | --- | --- | --- | --- | --- |
|  |  | **Non-T2D** | **T2D** | ***P* value** |  | **Non-T2D** | **T2D** | ***P* value** |
| *rs1978124* | *CC* | 139.2±3.4 | 139.7±6.0 | 0.425 |  | 4.21±0.32 | 4.17±0.26 | 0.180 |
|  | *TT+CT* | 138.9±4.0 | 141.2±5.1 | <0.001 |  | 4.26±0.21 | 4.15±0.27 | 0.001 |
|  | *P* value | 0.456 | 0.024 |  |  | 0.131 | 0.545 |  |
| *rs2048683* | *GG* | 139.1±3.6 | 139.5±5.6 | 0.327 |  | 4.24±0.31 | 4.14±0.27 | 0.001 |
|  | *TT+GT* | 139.4±3.5 | 141.9±5.3 | 0.001 |  | 4.18±0.16 | 4.20±0.26 | 0.710 |
|  | *P* value | 0.563 | 0.001 |  |  | 0.090 | 0.078 |  |
| *rs233575* | *CC+CT* | 139.6±3.7 | 140.7±4.6 | 0.095 |  | 4.25±0.24 | 4.22±0.32 | 0.474 |
|  | *TT* | 139.0±3.6 | 140.2±6.1 | 0.025 |  | 4.22±0.30 | 4.12±0.22 | <0001 |
|  | *P* value | 0.278 | 0.404 |  |  | 0.421 | 0.004 |  |
| *rs4646156* | *AA+AT* | 139.8±3.6 | 141.9±5.3 | 0.004 |  | 4.23±0.24 | 4.20±0.26 | 0.369 |
|  | *TT* | 139.0±3.6 | 139.5±5.6 | 0.230 |  | 4.22±0.30 | 4.14±0.27 | 0.003 |
|  | *P* value | 0.108 | 0.001 |  |  | 0.823 | 0.078 |  |
| *rs879922* | *CC+CG* | 139.6±3.4 | 141.0±5.2 | 0.002 |  | 4.21±0.28 | 4.20±0.25 | 0.699 |
|  | *GG* | 138.7±3.8 | 139.0±4.8 | 0.417 |  | 4.24±0.30 | 4.10±0.28 | <0.001 |
|  | *P* value | 0.082 | 0.013 |  |  | 0.315 | 0.004 |  |

**Table S7. Serum HsCRP levels in relation to different ACE2 SNPs among participants with or without T2D.**

| ***ACE2* SNPs** | | **HsCRP (mg/L)** | | |
| --- | --- | --- | --- | --- |
|  |  | **Non-T2D** | **T2D** | ***P* value** |
| *rs1978124* | *CC* | 12.1±13.0 | 10.6±12.5 | 0.283 |
|  | *TT+CT* | 10.2±9.2 | 14.5±14.8 | 0.023 |
|  | *P value* | 0.227 | 0.021 |  |
| *rs2048683* | *GG* | 11.3±12.4 | 13.0±15.3 | 0.233 |
|  | *TT+GT* | 12.7±10.6 | 11.7±11.1 | 0.582 |
|  | *P value* | 0.461 | 0.397 |  |
| *rs233575* | *CC+CT* | 12.8±10.2 | 12.9±12.3 | 0.972 |
|  | *TT* | 11.2±12.5 | 12.3±14.9 | 0.464 |
|  | *P value* | 0.382 | 0.726 |  |
| *rs4646156* | *AA+AT* | 12.3±10.3 | 11.7±11.1 | 0733 |
|  | *TT* | 11.4±12.5 | 13.0±15.3 | 0.257 |
|  | *P value* | 0.625 | 0.397 |  |
| *rs879922* | *CC+CG* | 10.2±8.1 | 13.8±12.8 | 0.003 |
|  | *GG* | 12.8±14.6 | 10.3±15.3 | 0.193 |
|  | *P value* | 0.064 | 0.055 |  |

**References**

1. Pan Y, Wang T, Li Y, Guan T, Lai Y, Shen Y, Zeyaweiding A, Maimaiti T, Li F, Zhao H *et al*: **Association of ACE2 polymorphisms with susceptibility to essential hypertension and dyslipidemia in Xinjiang, China**. *Lipids Health Dis* 2018, **17**(1):241.

2. Lang RM, Badano LP, Mor-Avi V, Afilalo J, Armstrong A, Ernande L, Flachskampf FA, Foster E, Goldstein SA, Kuznetsova T *et al*: **Recommendations for cardiac chamber quantification by echocardiography in adults: an update from the American Society of Echocardiography and the European Association of Cardiovascular Imaging**. *European heart journal cardiovascular Imaging* 2015, **16**(3):233-270.

3. Celeste F, Muratori M, Mapelli M, Pepi M: **The Evolving Role and Use of Echocardiography in the Evaluation of Cardiac Source of Embolism**. *J Cardiovasc Echogr* 2017, **27**(2):33-44.
